# Clinical Review, and Hospital Reports

**Published:** 1840-01-01

**Authors:** 


### Clinical Review.

Guy's Hospital Reports. No. IX. October, 1839. Edited by George Barlow, M.A. &c. &c. and James P. Babington, M.A. &c. &c.

The present Number is a worthy successor of those that have gone before—no mean praise, nor trifling recommendation. Sir Astley Cooper, as usual, is a contributor. The zealous Baronet is always on the alert to render his experience available to the purposes of science.

The contents of the Number are as follows:—

On the Dislocation of the Os Humeri upon the Dorsum Scapulæ, and upon Fractures near the Shoulder-Joint; by Sir Astley Cooper, Bart., F.R.S. D.C.L. (With Plates).—Case of Poisoning by Sulphuric Acid; by Alfred S. Taylor.—On Amputation; by Bransby B. Cooper, F.R.S.—Cases of Hernia; by Bransby B. Cooper, F.R.S. (With Plates).—Case of Empyema and Pneumo-Thorax; with Observations; by George H. Barlow, M.A. & L.M.—On the Physical Diagnosis of Incipient Phthisis; by H. M. Hughes, M.D.—Observations on the Pulse in Phthisis Pulmonalis; by William Augustus Guy, M.B.—On the Supposed Existence of Fluoric Acid as an Ingredient in certain Animal Matters; by G. O. Rees, M.D. F.G.S. &c.—Observations on the Incubated Egg; by Joseph Towne. (With Plates).—Report of Primary Syphilitic Cases; by Charles Aston Key.—On the Preservation of Subjects for Anatomical Purposes; by B. G. Babington, M.D., and G. O. Rees, M.D.

It would be inexcusable did we not pay our first respects to Sir Astley.

### 1. On the Dislocation of the Os Humeri upon the Dorsum Scapulæ, and upon Fractures near the Shoulder Joint. By Sir Astley Cooper, Bart. F.R.S. &c. &c.

When Sir Astley published the last edition of his Work on Dislocations and Fractures, he had not enjoyed an opportunity of examining the appearances on dissection of a dislocation on the dorsum of the scapula. Such an opportunity has now occurred, and Sir A. contributes the particulars which he has learnt to the profession.

*Case.* "I was sent for by Mr. Complin, a gentleman residing in Bishopsgate-street, in the city of London, to examine his left shoulder: and I found a very obvious luxation of the os humeri upon the dorsum scapulæ, as was evinced by the depression below the acromion, by the absence of the head of the bone from its natural situation, by a depression by which the fingers could be admitted into the glenoid cavity between the head of the os humeri and the coracoid process, but more especially by the projection of the head of the os humeri upon the back part of the glenoid cavity of the scapula, and upon its inferior costa, as well as by its filling the space between that costa and the spine of the scapula.

I advised an extension; but he had already suffered so much from attempts at reduction, that he was full of apprehension at the least motion of his shoulder, so that I no longer urged it, and he retained the appearances of the displacement to his death. Dr. Cobb had seen him. Mr. Key also had examined his shoulder with care; and his son, who is a surgeon in Charterhouse-square, has given me the particulars of the case, in a letter which I received from him:

'Mr. Complin was 52 years of age, and had been the subject of epileptic fits. One of these, which was particularly severe, occurred one morning whilst he was in bed, and in his violent convulsive strugglings his shoulder became dislocated. Mrs. Complin, who was with him, is confident that he neither fell from his bed nor struck his shoulder, during the fit: and that the appearances which I have described, and which remained until his death, were the result of muscular efforts alone, was the firm belief of Dr. Cobb, Mr. Key, and myself. Beside the unusual mode in which the accident happened, there were two other circumstances peculiar in the case. The one was, that the head of the bone could be, by extension, drawn into its natural situation in the glenoid cavity; but so soon as the force ceased to be applied, the head of the bone slipped again upon the dorsum scapulæ, and all the appearances of dislocation were renewed. The second peculiarity consisted in a sensation of crepitus as the bone escaped from its socket, so as to lead to a belief that the edge of the glenoid cavity had been broken off; and this symptom led to a belief that the bone so readily slipping from its situation, when restored to its natural cavity, arose from the imperfection of the articular surface of the glenoid cavity.'

The patient was unable to use, or even to move the arm to any extent; nor could he, by his own efforts, elevate it from his side: and although he lived seven years after the occurrence of the epileptic fit, he never recovered the use of the limb."

After his death, the scapula and os humeri were sent to Sir Astley, who found the state of the muscles and the situation of the bones to be the following:—

The head of the os humeri was placed behind the glenoid cavity of the scapula; and it rested upon the posterior edge of that articulatory surface, and upon the inferior costa of the scapula, where it joins the articulation. When the scapula was viewed anteriorly, the head of the os humeri was placed in a line behind the acromion, but below it; and a wide space intervened between the dislocated head of the bone and the coracoid process, in which the fingers sunk deeply towards the glenoid cavity of the scapula.

When viewed posteriorly, the head of the os humeri was found to occupy the space between the inferior costa and spine of the scapula, which is usually covered by the infra-spinatus and teres-minor muscles. The tendon of the subscapularis muscle and the interior portion of the capsular ligament had been torn at the insertion of that muscle; but the greater part of the posterior portion of the capsular ligament remained, and had been thrust back with the head of the bone, the back part of which it enveloped.

The supra-spinatus muscle was put upon the stretch; the subscapularis diminished by want of action; and the infra-spinatus and teres-minor muscles were shortened and relaxed, as the head of the bone carried their insertions backwards. The tendon of the long head of the biceps muscle was carried back with the head of the bone, and elongated; but it was not torn.

As to the changes in the bones, the head of the os humeri and the outer edge of the glenoid cavity of the scapula were in direct contact, the one bone rubbing upon the other when the head of the os humeri was moved; and this accounted for the sensation of crepitus at the early period of the dislocation, as there was no fracture. The glenoid cavity was slightly absorbed at its posterior edge, so as to form a cup, in which the head of the bone was received; and this latter bone, and the articular cartilage, had been in some degree absorbed where it was in direct contact with the scapula, as well as changed by attrition during the seven years the patient lived.

The surface of the original glenoid cavity, instead of being smooth and cartilaginous, was rough and irregular, having elevations at some parts, and depressions at others. The extremity of the acromion was sawn off, to look for any little fragment of bone which might have been broken off; but not the smallest fracture could be perceived.

"The dissection," continues Sir Astley, "of this dislocation explained well the whole of the circumstances.

In the usual dislocation backwards, or on the dorsum scapulæ, the bone, when reduced to its proper situation, remains there; and only requires a roller, and rest for a few hours, to prevent the future escape of the bone from the glenoid cavity; and the muscles, recovering their tone, fix it in its natural cavity. But in this case, as the tendon of the subscapularis and the capsular ligament had been torn from the smaller tubercle of the head of the os humeri, and the bone was consequently drawn back by the action of the infra-spinatus and teres-minor muscles, there was no support given to the head of the bone, when returned into its cavity; but so soon as it was replaced, it was drawn back by the action of the two posterior muscles, and all the appearances of dislocation reproduced.

The bandage required to keep the bone in its place should be placed on the fore-part of the chest, and behind the shoulders, to keep the head of the bone forwards; and a pad ought to be placed behind the head of the bone, to prevent it from slipping from its cavity: so that the bandage would be the reverse of the common stellate, which crosses upon the back; but this should be made to cross upon the anterior part of the chest: and to this bandage a sling must be added, to support the elbow, and to keep it back."

Sir Astley goes on to state, that, since he last wrote on dislocations of the shoulder, several cases of dislocation on the dorsum scapulæ have come to his knowledge. The accident usually happens by a violent push of some opposing body whilst the arm is advanced, and the head of the bone is thrown much further backward than in Mr. Complin's case; and probably the posterior portion of the capsular ligament is torn.

"In these cases, I have seen the common means of reduction employed, viz. fixing the scapula by a band, and drawing the arm outwards from the body; but the reduction was slowly performed, and was with difficulty effected. I was therefore pleased with the following case; in which the reduction was easily effected, after twenty-three days, without any forcible or complicated plan.

A gentleman who was staying in Essex got into a scuffle with a man; and pushing him violently from him, he dislocated the os humeri backwards. He consulted a surgeon in the place in which he was staying at the time of the accident; who advised his going to London, to consult an hospital surgeon. He followed this advice on the seventeenth day after the accident; and the surgeon, to whom he applied, tried to restore the bone to its place, but it resisted all his efforts. On the twenty-third day he applied to me; and the nature of the accident was sufficiently obvious, as the projection of the head of the bone could be readily felt; and when the elbow was rotated, the head of the os humeri could be perceived rolling upon the dorsum scapulæ. As he called upon me at my own house, I had no means of making the usual extension at the moment: but I placed the patient upon a chair; and bending his elbow at right angles, I raised his arm, and carried it behind his neck, so as to bring the hand across the back of the neck to the opposite shoulder; then forcing the elbow back, and pressing upon the head of the bone, I pushed it under the inferior costa of the scapula, and it instantly returned into the glenoid cavity. Mr. Bransby Cooper was present at the reduction of the dislocation.—Other means, however, will succeed."

A man came to the surgery of Guy's Hospital; and Mr. Tallent, dresser of Mr. Bransby Cooper, placed the patient upon a chair, put his knee in the axilla, whilst his fellow-students extended the arm from the wrist under his thigh, and the bone was reduced.—Mr. Dunn, surgeon in Blackfriars, also succeeded easily, by fixing the scapula, and extending from the wrist, in the usual manner of reducing a dislocation in the axilla.

In a case attended by Messrs. Whittaker and Key.

*Case.* A stout man fell down on his shoulder, and dislocated the head of the humerus on the dorsum scapulæ, where the head of the bone could be seen and felt. The elbow could be brought to the side, or raised on a level with the acromion. Rotation outwards was entirely impeded, in consequence of the subscapularis being stretched, all motion of the limb giving him extreme pain; which was referred to the lower part of the deltoid muscle, in the direction of the articular nerves, which were probably injured by the pressure of the head of the bone.

"At first, we placed our patient on a chair; and while Mr. Whittaker fixed the scapula, I carried the elbow upwards and backwards, in order to throw the head of the bone forwards into the glenoid cavity, but to no purpose. All that we gained by this measure was a slight advance of the bone, and reducing him to a state of syncope. In this state, we laid him upon his back; and fixing his scapula by the heel in the axilla, drew the arm downwards; and by extension, continued for a minute or two, succeeded in reducing it."

### II. OF FRACTURES OF THE HEAD AND NECK OF THE OS HUMERI.

Sir Astley has arranged and communicates the results of his experience on these troublesome and frequently perplexing accidents. He has found them of various kinds:—

First—Dislocations of the os humeri into the axilla, with fracture, and detachment of the head of the bone, which is thrown on the inner side of the inferior costa of the scapula.

Secondly—Fractures through the neck of the bone, at the tubercles; in which the head of the humerus is broken off, but it remains in the glenoid cavity. This fracture occurs at the epiphysis, or anatomical neck of the bone.

Thirdly—A fracture below the articulation, between it and the insertions of the pectoralis-major, latissimus-dorsi, teres-major, coraco-brachialis, and deltoid muscles. This part has been called the surgical neck of the bone.

### 1. *Of Dislocation and Fracture.*

A person, says Sir Astley, falls, pitches with violence upon his shoulder, or a heavy-laden carriage passes over it: by the first impression of the accident, the os humeri is dislocated; and by a second, the neck of the bone is broken, and the head is detached, and lodges in the axilla.

The signs of this accident are, the usual symptoms of dislocation of the os humeri into the axilla, the head of the bone being there felt; but with somewhat less hollow below the acromion, and behind the deltoid muscle, because the broken extremity of the shaft of the bone quits the fractured head, and becomes situated in the glenoid cavity of the scapula. Upon rolling the arm, the broken shaft of the bone can be perceived to move under the acromion. There is little power of motion; and considerable pain, not only in the shoulder, but in the arm and hand.

The head of the os humeri can be felt in the axilla, when the arm is raised and the surgeon's fingers are thrust into the axilla: but when the arm is rolled at the elbow, the head of the bone remains entirely unmoved, or very little obedient to the motion of the elbow.

There is, in some cases, but not always, a distinct crepitus; but more frequently a gristly feeling, from friction of the broken neck of the bone against the glenoid cavity and its cartilaginous covering.

The broken end of the os humeri is drawn somewhat forwards; but is easily pushed into the glenoid cavity; from which, unless it be supported, it is again drawn by the pectoralis and coraco-brachialis muscles.

The arm, measured from the acromion to the elbow, is shorter than the other.

As great violence has occasioned this accident, the parts are much obscured, by the effusion of blood, and by the inflammation which speedily follows: but, for the first three hours, the muscles are so lax, that, but for the pain it occasions, considerable motion of the limb may be produced.

Sir Astley believes that he has seen many of these cases. He has had an opportunity of dissecting three.

In the *first*, (the patient died some time after the accident) the deltoid, teres-major, and coraco-brachialis did not appear altered. The supra-spinatus was somewhat wasted, as was the teres-minor; and their colour was fainter than natural. The infra-spinatus was put upon the stretch. The subscapularis passed over the head of the bone, and adhered to its cartilaginous surface.

The capsular ligament had been torn, under the subscapularis tendon; but every other part was entire.

The head of the os humeri had been broken off; and was found in the axilla, behind the coracoid process of the scapula, and it was strongly united to the inner side of the scapula.

The fractured neck of the shaft of the os humeri was seated in the glenoid cavity of the scapula, widely separated from the head of the bone; and the broken os humeri formed a new and good articulation with the glenoid cavity, with a capsular ligament over it, which was in part newly formed. A ligamentous matter passed from the broken end of the os humeri to the inner surface of the glenoid cavity of the scapula.

The edge of the glenoid cavity remained; but its surface was rather ligamentous, than smooth and cartilaginous.

The greater tubercle of the os humeri had become enlarged; the tendon of the biceps remained in the bicipital groove; and the tubercles were connected with the broken extremity of the shaft of the os humeri, and not with its head.

In the *second*, (the accident had also happened long previously), the head of the os humeri was dislocated into the axilla, and broken from the shaft; and it remained upon the inner side of the inferior costa of the scapula, to which it was firmly united.

The tubercles of the neck of the os humeri were broken off with the head of the bone; and the fractured extremity of the neck of the os humeri was placed in the glenoid cavity of the scapula.

The underhand motions of the shoulder were restored; but the elevation of the bone, beyond a right angle, was strongly resisted, and even with difficulty could be accomplished in the dead body.

In the *third* case, the head of the os humeri was broken off; and it rests in the axilla, upon the subscapularis muscle, and on the inner side of the inferior costa of the scapula.

The infra-spinatus and teres-minor muscles were much wasted, on account of the arm having its motions in a great degree destroyed.

The supra-spinatus and subscapularis muscles were less diminished; as what motion remained was performed principally by them.

The tendon of the biceps flexor cubiti had been torn, but its remains adhered to the bicipital groove.

The broken upper extremity of the os humeri was lodged in the glenoid cavity of the scapula, and had formed there a ligamentous joint.

The head of the humerus had a new capsular ligament, to which the axillary artery adhered.

The head of the bone was not joined to the scapula, as in the two former instances; but it was united, by a small process of bone, to the os humeri, at the part at which it had been broken off.

The head of the bone was placed on the sternal side of, and a little below the level of, the coracoid process of the scapula.

In enumerating the diagnostic signs which distinguish this from simple dislocation into the axilla, Sir Astley would observe that the fall and depression of the shoulder is less striking than in that accident, the shaft of the bone filling up the glenoid cavity.

That still the head of the os humeri can be distinctly felt in the axilla; and, that as it does not roll when the os humeri is rotated from the elbow, this becomes the principal diagnostic mark.

That a grating sensation can generally be felt, and sometimes a very distinct crepitus, especially if the elbow be raised outwards during the rotation of the arm.

That the upper broken extremity of the os humeri can be felt advancing to the coracoid process; but that it is easily returned into the glenoid cavity, and that it there rotates with the arm, but easily again slips forward.

That the accident which produces it is more violent than that by which simple dislocation in the axilla is produced; and there is, therefore, a greater appearance of contusion, more swelling, and more pain.

Speaking of the treatment, Sir Astley says:—extension is of no further use, than to bring the broken shaft of the os humeri into the glenoid cavity, where it forms an useful articulation; but no extension, however violent, disturbs the broken head of the bone, for no proper force could bring it into the glenoid cavity of the scapula. If reduction be ever effected, it will probably be by an extension with the heel or knee in the axilla.

To keep the broken end of the shaft of the bone in the glenoid cavity, a pad must be put in the axilla, to thrust it outwards: a clavicular bandage must be used, and the arm be supported in a sling.

But let the surgeon do what he will, the head of the bone will probably remain in the axilla, and the upper motions of the arm will be in a considerable degree lost.

And with that frankness which has ever distinguished him, Sir Astley adds:—"These cases should teach the members of our profession to be kind, generous, and liberal towards each other; and not to impute to ignorance or inattention that which is the result of a generally incurable accident. It too often happens, that, when every trial has been made to restore the parts, and without success, the patient goes to some other surgeon, to whom he shews his arm, and points out its uselessness and want of motion. A jealous and illiberal medical man might say, 'Yes, this is a dislocation which has not been reduced: I wish I had seen it at first; but now it is too late for a successful attempt to replace it.' However, every intelligent well-informed surgeon will *now* confess, that no knowledge, or exertion of skill, could have prevented the deformity and loss of the natural motion which results from this formidable accident."

2. *Of Fracture through the Tubercles.*

This fracture, Sir Astley tells us, happens at the junction of the head of the os humeri with the tubercles, at the part at which the capsular ligament is fixed, and where, in young people, the epiphysis is placed.

It is a very frequent occurrence in young people: it sometimes, though more rarely, happens in the old: in middle age it seldom occurs.

In children, it is the result of falls upon the shoulder; or it happens from a sudden or unexpected push of the arm, which it is unprepared to resist.

Sir Astley has seen it complicated with fracture of the clavicle. He gives as signs of the accident:—

The head of the bone remains in the glenoid cavity of the scapula; so that the shoulder is not sunken, as in dislocation.

When the shoulder is examined, a projection of bone is perceived upon the point of the coracoid process; and when the elbow is raised and brought forwards, this projection is rendered particularly conspicuous.

By drawing down the arm, the projection is removed; but it immediately re-appears upon giving up the extension, and the natural contour of the shoulder is lost.

The motion of the shoulder is painful; and the child cannot raise the arm but with his other hand: the elbow is with difficulty removed from the side; and the arm is obliged to be supported, either by the patient himself, or by aid of another.

Sir Astley has known the accident mistaken for dislocation. The point of the broken bone is felt at the coracoid process, and this is supposed to be the head of the os humeri; but, with care, the head of the bone can be felt still filling the glenoid cavity. When the elbow is rolled, the head of the bone does not obey its motion.

A slight extension draws the broken point of the bone into the natural position, beneath the head, from which it has been separated; but it always immediately re-appears, when the extension is lessened or removed.

Upon the dissection of these cases in the young, the head of the os humeri is found broken off at the tubercles, but it remains in the glenoid cavity.

A great quantity of ossific matter is thrown out from the periosteum and fractured neck of the shaft of the bone, but very little from the broken head. A cup of bone is formed upon the fractured neck of the shaft, in one of Sir Astley's preparations, which supports the head of the bone, so as to prevent the neck separating from it. A slight union is produced by the cancellated structure; the principal callus being formed on the outer surface, and it encases the bone.

Sir A. Cooper relates a case which occurred in the practice of a very able surgeon, Mr. Webster, of the Edgeware Road. The patient was 77 years of age, a period of life at which such an accident is necessarily rare.

The best mode, says Sir Astley, of treating these accidents consists, in the young, in applying a splint on the fore and back part of the arm, binding it on by a roller; placing a pad in the axilla, and using a clavicular bandage; supporting the hand, but not the elbow, in a sling; as, if the elbow be raised, the broken end of the bone projects forwards.

In old persons, the injury is more severe, and the force producing it is violent: it therefore becomes necessary to reduce inflammation, and to apply leeches and evaporating lotions; to observe perfect rest at first: and, after some time, the same treatment, as to bandages, may be pursued as in the young.

In both the old and the young, passive motion is to be employed so soon as the union is effected: which, in youth, is in a month; but it requires from two months to twenty weeks, in age.

### 3. *Of Fracture of the Humerus at its Surgical Neck.*

The bone is broken between the tubercles and the insertion of the pectoralis-major, coraco-brachialis, latissimus-dorsi, teres-major, and the deltoid muscles.

In this case there is great deformity of the bone: the head, neck, and tubercles remain in the glenoid cavity, with part of the shaft of the bone connected with them; but the broken end of the shaft is drawn forward and upward, under the pectoralis-major muscle.

When the elbow is thrust upwards, the broken extremity of the bone projects on the inner side of the coracoid process of the scapula; and it sinks when the support of the elbow is removed.

When the arm is rotated at the elbow, the broken end of the lower part of the bone is felt to roll.

There is no marked depression under the acromion; or but very little, if any; and then it happens from the deltoid muscle being dragged down.

The motion of the shoulder is extremely painful; and the patient has generally one or more fingers affected, sometimes contracted, at others painful only; and this depends upon one or other of the nerves of the axillary plexus being irritated by a part of the bone.

The elbow admits of being moved in all directions; for there is much less confinement of the arm at the shoulder than in the other accidents of that part; however, the movements are very painful.

The diagnostic signs of this accident are found in the head of the bone being in its cavity; in its being unaffected by the rotation of the elbow; in the point of the fractured neck being felt under the pectoral muscle; and in the surgeon being able to move the arm much more freely than in the other fractures of the neck of the bone.

Such is Sir Astley's account of this variety of accident. He relates a case which occurred to Mr. Blenkarn of Bermondsey, and which seems to have been managed with equal skill and success.

In the teatment, adds Sir Astley, the splints, the clavicular bandage, and the pad in the axilla, are required: but, above all, it is necessary to permit the arm to hang by the side, unsupported at the elbow; so as to let the weight of the arm be a constant source of extension upon the broken end of the bone. He subjoins:

"Mr. Tyrrell informs me, that in a case of fracture at the tubercles, he found that the bone best maintained its natural position by its being raised and supported at a right angle with the side, by a rectangular splint, a part of which rested against the side, whilst the arm reposed upon the other part; and until he made use of this plan, he could not succeed in removing the deformity, or in keeping the bone in its place.

Mr. Guthrie has given an interesting account of a curious case of fracture of the neck of the bone, in the Medico-Chirurgical Review, Vol. VIII. p. 289:—

A man fell from a ladder, and received a severe injury on his shoulder. Mr. Guthrie saw him about three hours after the accident; and the most remarkable and striking appearance was a fold or pucker of the skin, the size of half-a-crown, situated over the pectoral muscle. A hard substance could be felt below; and extending, above it, to the coracoid process. He says, 'I decided that it was a fracture, not a dislocation. The arm was moveable in every direction; and the elbow could be brought close to the side.' After the swelling had subsided, he says, 'I decided that the bone had been broken at its anatomical neck, and forced through the pectoral muscle.'

He found that he could bring the bone down to its natural situation, as to length; but it would not exactly remain in its proper place.

The man recovered, with a good use of his arm; so that the case terminated very favourably."

This paper of the worthy Baronet's deserves the attention of our readers.

### III. On the Preservation of Subjects for Anatomical Purposes. By B. G. Babington, M.D. and G. O. Rees, M.D.

It is but too notorious, that the Anatomical Bill has worked and been worked badly, and is, every year, working and being worked worse. If we go on, in an arithmetical ratio, as we have begun, the inspector will soon have his salary for nothing, for there are no dissections to inspect, and anatomical lectures will be limited to the description of the vital *principle*, in the absence of anything more tangible. Impressed with a laudable desire to make the most of the few bodies that the zeal and the exertions of the inspector can procure, Drs. Babington and Rees have tried many experiments on the best method of preventing putrefaction. We need say nothing of the methods that failed. But they bethought themselves at last of creasote and pyroxylic spirit. They first injected rabbits, with excellent success. Creasote was dear—pyroxylic spirit cheaper, and the pickling of the rabbits having answered so well, they determined to try what could be done with a human subject. We quote what follows:—

"On the 15th of May last, a convict at Woolwich, 23 years of age, died of inflammation of the bowels; and, on the 18th, his body was sent, by order of the Inspector of Anatomy, to Guy's Hospital, for dissection. It was neither œdematous, nor in a state of decomposition; and although the integument was somewhat fat, it was, upon the whole, in a fair condition for anatomical purposes. On the 21st, a gallon of pyroxylic spirit was injected into the aorta; and the body was placed in a water-tight shell, or trough, made of slate, and loosely covered with a wooden lid. This trough was deposited in a cellar, the stone floor of which was about two feet below the surface of the ground. On the 29th, the lid was removed, for the first time, and the body was found to be perfectly fresh. On this occasion, the flesh of the extremities was remarked to have become somewhat firmer than when the injection was first made. From the 29th of May to the 12th of June, the subject was examined, by removing the lid of the trough every two or three days; and no change was perceptible until the latter date. At that time, the only sign of alteration was the appearance of two or three brown streaks—evidently veins—on the inside of the thighs; and a separation of the cuticle of the hands from the true skin, which began to assume a greenish hue. Every other part of the body was perfectly preserved, and of natural colour. There was no putrid odour on opening the lid of the trough, but the characteristic smell of the pyroxylic spirit was in some measure passing off. An incision into the middle of the right thigh, such as would be made in operating for popliteal aneurism, shewed that the fat, muscles, blood-vessels and nerves were in a complete state of preservation. It should be observed, that ever since the injection of the subject the weather had been that of established summer; and that a second body, received from Woolwich, was so decomposed in three days after its arrival as to be totally unfit for dissection. On the last examination, as well as on two or three previous occasions, fluid was observed to occupy the bottom of the trough, and this it was thought adviseable to remove; it was likewise determined to throw another quart of pyroxylic spirit into the aorta.

On the 24th of June, the body was removed to the dissecting-room, and placed on the table, for the purpose of being thoroughly dissected. With the exception of a greenish appearance on the outer part of the left thigh, and the brown streaks already mentioned, it appeared, when brought into the light, perfectly preserved. The skin on the back of the hands, instead of putrifying, had dried, and become transparent; while the greenness of the left thigh proved on incision, to be quite superficial. The dissection was undertaken by eight gentlemen, and completed by the 13th of July; and it is testified by them all, that every anatomical purpose was as fully answered as if the subject had been quite recent. The various parts, on being laid open, were of natural colour and of firm texture. The tendons and ligaments were silvery and white, and the nerves had lost none of their tenacity. The pectoral muscles alone formed an exception to the natural colour which was elsewhere maintained: this appeared to be attributable to the macerating effects of a wetted cloth that had been laid upon the breast, to prevent evaporation through the aperture by which the injection had been accomplished. The parts which were exposed by dissection gradually dried; changing, in the course of a day or two, to a dark colour, and instead of putrifying, becoming hard. The brain, although it had retained its form, was soft, semi-putrid and unfit for demonstration: it must be borne in mind, however, that had the head been opened six days after death—at which period the subject was injected—this probably would have been the case. With the above exception, the viscera were all perfectly preserved: in proof of which, one of the kidneys, appearing, in colour and consistence, quite recent, was removed in the beginning of July, and, after maceration in warm water, in the usual manner, was injected with wax. This experiment was made in order to ascertain whether the spirit produced thickening, or any other alteration, in the inner coat of the blood-vessels; which was found not to be the case, as the wax had fully penetrated the tissues of the organ.

Of the gentlemen engaged in the dissection of this subject, one complained that he at first suffered headache from the odour which it exhaled; and some, who were not so engaged, considered this to be more disagreeable than that of putridity. The same opinion is sometimes expressed with respect to the odour of parts that have been macerated in spirit of wine. Some allowance in favour of the pyroxylic spirit should be made on the score of novelty; and since its vapour is not poisonous nor injurious, any more than that of spirit of wine, it is to be presumed that the student would soon become accustomed and reconciled to it. In a first trial, upon the human subject, of the antiseptic powers of this fluid, a natural desire existed, on our parts, of watching its progress, and of noting such changes as might gradually occur. This led to the necessity of opening frequently the lid of the trough: and it has already been remarked, that this by no means accurately fitted the trough itself. The pyroxylic spirit being of a very volatile nature, it is obvious that its preservative qualities were much diminished by this proceeding. It is, therefore, not too much to expect that in an air-tight vessel a subject thus prepared would not exhibit even those superficial changes which took place in this instance, and would be preserved for an indefinite period.

The advantages of employing pyroxylic spirit are, 1st, its extreme fluidity, in consequence of which it may be thrown into the minutest vessels. 2dly, its freedom from colour. 3dly, its cheapness; for a gallon is sufficient to inject a full-sized subject: and even with the present limited manufacture of it, it is only half the price of alcohol; while it possesses infinitely greater antiseptic powers, and is, in common with that fluid, miscible with water, in all proportions. 4thly, its innocuous nature, and its freedom from any corrosive action upon steel instruments. We are not aware that there is any material disadvantage in its employment: the odour, it must be admitted, is more or less disagreeable to different individuals, but not so much so, to the generality of persons, as that of the putridity which it serves to prevent. Of this fluid, which must not be confounded with pyro-ligneous acid, or with pyro-acetic spirit, a full account may be found in the 'Annals of Philosophy,' N.S. viii. 69. That which we employed was procured from Morson's, in Southampton Row; and it may be had from any operative chemist."

The plan must be tried extensively, and we hope that it will succeed so well as this account might lead us to anticipate. Much credit is due to the experimenters.

### IV. APPEARANCES AFTER DEATH IN A CASE OF POISONING BY SULPHURIC ACID.

These are detailed by Mr. Alfred Taylor. The patient was 40 years of age —took about a wine-glassful of sulphuric acid—and died twenty-five hours afterwards.

*Dissection.*—This took place three days after death. The body was stout, and well-formed; free from marks of lividity or ecchymosis. Decomposition had already commenced about the head and abdomen. There were no traces of the action of the acid on the exterior of the face and neck. The membrane covering the tongue and palate was white, soft, and thickened: on removing this, the parts beneath were intensely reddened. The membrane lining the pharynx and œsophagus was corroded throughout its length. It was of an ash-grey colour, disposed to longitudinal rugæ, between which a brownish-coloured liquid was interposed. The membrane was not particularly softened: it resisted the pressure of the finger. The epiglottis, and the parts about the glottis, had become affected by the acid; but the larynx itself had escaped.

On opening the abdomen, the stomach was seen lying collapsed: externally, towards the cardia, it was a deep-red colour, intermixed with black striæ. There was no perforation of its coats, which were pretty firm. The small intestines, especially the duodenum, and upper part of the jejunum were inflamed. On laying open the stomach, it was found to contain no liquid, but a brownish-black pasty matter. This was washed off, and reserved for analysis. The mucous membrane was universally blackened; and raised into thick prominent cords or bands, which were disposed parallel to the greater curvature between the cardia and pylorus. Towards the pyloric extremity, the black discoloration, and, with it, the rugose condition of the mucous membrane, ceased. The coats of the organ were not perceptibly softened; and even the blackened parts resisted the pressure of the finger, having a hard corrugated feel. On stretching the stomach slightly, the black rugæ were found to be bordered, at the margin, with a deep crimson-red colour, from the inflamed mucous membrane beneath. This black discolouration was probably due, partly to the carbonizing action of the acid, and partly to its effect on the blood contained in the vessels of the mucous membrane.

The mucous membrane of the small intestines was inflamed, and the duodenum presented the same black discolouration as the stomach. The liver was of a deep livid colour, and congested. The lungs were of a dark grey, but not particularly full of blood. The heart and large vessels connected with it, were much distended with blood, mostly in a coagulated state. The lining membrane of the descending aorta was of a bright scarlet colour. The other organs presented no appearance requiring notice.

The contents of the stomach, with the organ itself, were digested in water; and a deep brownish-coloured liquid was obtained. When the insoluble matters had subsided, this liquid was filtered and tested. It was faintly acid. It gave a precipitate with nitric acid, probably albumen; but there was not the slightest indication of sulphuric acid, either free or combined, on applying the usual re-agents. The poison had therefore become effectually removed by vomiting and purging; aided, perhaps, by the action of the magnesia, which had been freely exhibited.

### V. Dr. Rees on the Absence of Fluoride of Calcium, as an ingredient in Animal Structures.

After noticing the positive announcement of Berzelius, that fluoric acid may be detected in human teeth, bones, and urine, Dr. Rees remarks:—

"The experiment which the Baron recommends, in order to obtain corrosion from bone-earth, is, to distil equal parts of strong sulphuric acid, and water upon it, until the measure of water is brought over. He states, that the distilled liquor, if evaporated in the glass receiver, will produce a corrosion.—I repeated this experiment, using 100 grains of bone-ash, and an ounce of the acid mixture; but could obtain no action on the receiver, by evaporating the distilled liquor; nor was there any corrosion or opacity produced on any part of the apparatus.

During the evaporation of the last portions of the liquor, dense white fumes appeared; and there was some difficulty in vapourizing the whole of it. On neutralizing a portion with ammonia, and testing it with nitrate of silver, a yellow precipitate of phosphate of silver was thrown down. A further examination shewed the presence of sulphuric acid, and traces of hydrochloric acid. I was much surprised to find phosphoric acid in this result of aqueous distillation, as the heat had not been urged during the process; for I had considered that acid as of too fixed a nature to volatilize with water at so low a temperature. It appeared to me now, that the presence of phosphoric acid, in this distilled liquor, might be a source of fallacy in the above experiment for establishing the presence of fluoric acid as a constituent of human bone; for it is a well-known fact, that phosphoric acid, if heated on glass of inferior quality till it volatilize, will act upon it with considerable energy; and all the animal substances in which fluorine has been said to exist, are particularly rich in phosphoric acid;—thus the ashes of ivory, of human bone, and the enamel of teeth, as also the precipitate obtained from urine by means of lime-water, are all of them composed, in very great part, of phosphate of lime. Mr. Richard Phillips has mentioned (in the Annals of Philosophy, Vol. V.), that when the water contained in uranite is driven off from the powdered mineral, a portion of the phosphoric acid is volatilized with it; the heat used being that of a common spirit lamp. This is the only fact with which I am acquainted (with the exception of my own observation), to shew that phosphoric acid will volatilize with water. The heat used in Mr. Phillips's experiment was, most probably, considerably higher than that which I applied. There seems no doubt that phosphoric acid is much more volatile than it has heretofore been supposed.

Having failed in detecting fluoric acid in human bones, I determined on testing for its presence in the enamel of teeth, in recent ivory, and in the precipitate obtained from urine by the addition of lime-water. Two different specimens of ivory (tusks of the elephant) gave no evidence of the presence of fluoric acid, when carefully tested, either before or after calcination; and I was equally unsuccessful with the enamel of human teeth, and the precipitate from the urine.

In these experiments, when I had failed in acting on the glass, I always found that the addition of 0.3 grains of fluoride of calcium to the experiment produced a strong and indelible mark on the surface of the glass test-plate. I mention 0.3 grains, because it will always be found sufficient to produce a most unequivocal corrosion: though I obtained satisfactory results by the addition of a much less quantity.—I have had only one opportunity of examining fossil ivory; and in that instance, I could not ascertain its locality: on submitting it, however, to the test used for recent ivory, bone, &c., I obtained immediate action on the glass.

In conclusion, I must express my firm conviction, that fluoride of calcium, as an ingredient in fossil ivory, must be regarded as an extraneous matter introduced by the partial mineralization of the animal substance;—that no such constituent exists in recent ivory, the enamel of teeth, human bone, or urine;—in fact, that fluoride of calcium should be expunged from the list of the constituents of animal substances."

### VI. ON THE PHYSICAL DIAGNOSIS OF INCIPIENT PHTHISIS. By H. M. HUGHES, M.D.

Dr. Hughes admits the difficulty and insists on the advantage of physical diagnosis, or of correct diagnosis assisted by physical investigations, in the early stage of phthisis. He attempts to contribute to our means of attaining the precision we so much desire.

The local signs, he says, of tubercular irritation may, like those of advanced phthisis, be obtained by three modes of investigation—inspection, auscultation, and percussion. And he observes that it may be premised, that none of the physical signs of either the early or advanced stages of phthisis are pathognomonic; that their value depends almost entirely upon their existing primarily in certain parts of the lung, and progressively extending to the remainder of the organ; that the physical diagnosis of the disease, in fact, results from comparison of the phenomena observed in the examination of the different regions of the chest. The importance of this comparison arises from the well-ascertained and acknowledged facts, that phthisis almost uniformly commences in the superior part of the lung, and gradually proceeds downwards; and that the disease, in the great majority of cases, does not affect both lungs equally, but appears earlier, or advances more rapidly, on one side than the other. Hence it arises, that when tubercles are deposited throughout the entire extent of the organ, as in some very uncommon cases, the diagnosis derived from physical investigations entirely fails.

*Inspection.*—To practise this mode of examination, it is of course necessary that the upper part of the chest should be bare, and also that the patient should be quite at ease, should use no muscular exertion, and should, in whatever position, be perfectly straight. It is desirable also, that the patient should be placed opposite to the light; that he should recline, with his back supported in bed or a high-backed chair; that his head and shoulders should be thrown back. When thus situated, it is not very uncommon that, previously to any investigation, there may be observed, in persons naturally well formed, a difference in the contour of the two infra-clavicular regions; one being full and rounded, the other comparatively depressed and flat. The difference, it is true, is often very slight; but it should not, therefore, be disregarded: for though, when existing alone, it is of little value as an indication, it may be of importance, as a corroborative evidence, when viewed in connection with other signs of the disease. On now directing the individual to take a deep inspiration, there is frequently observed a very obvious variation in the amount of expansion of the two sides; one being elevated, as in health; the other being comparatively motionless. This difference of expansion may be sometimes better appreciated, and on all occasions may be confirmed, by the sense of touch. Thus, by placing the expanded hands at the same time on the corresponding sides, and again telling the patient to take a full inspiration, the unequal expansion frequently becomes increasingly evident.

This is very good advice, but Dr. Hughes must be aware, and indeed admits, that there is no novelty in it. Every good physician employs this means of diagnosis as a matter of course, and frequently derives great advantage from it. We confess ourselves sceptical of the sub-clavicular flattening as an *early sign* of phthisis. Dr. Hughes observes:—

"It may be necessary to mention, that an appearance of sinking may sometimes exist on one side, depending on partial emphysema and consequent increased roundness and fulness of the other. The diagnosis, however, will be evident on further investigation; as not only will the larger side be unnaturally resonant on percussion, compared with other parts of the chest, but will evidently expand less than the opposite side on deep inspiration.

On referring to Dr. Stokes's valuable publication on diseases of the chest, after this paper was nearly written, I was much gratified to find that he had observed the early and frequent flattening of the infra-clavicular regions in phthisis. For the purpose of accurately determining the difference of the two sides, he recommends admeasurement in the antero-posterior direction, by calipers. I have not adopted this mode of ascertaining the point under consideration; as I had not heard, and, I must confess, had not thought of the practice. But I may observe, in reference to admeasurement of the chest, that if alone trusted, or if regarded as superior in accuracy to the eye, it may often lead to error. I have seen this very strikingly exemplified in cases of empyema; in which, though the eye distinguished an evident bulging of one side, it was found, on accurate admeasurement, to be very little or not at all larger, and in one case even less than the healthy side. Dr. Williams has also, in his admirable little work, mentioned, though very briefly, the difference of expansion in the two sides of the chest at an early period of phthisis; and Andral has observed the occasional occurrence of flattening or depression."

*Auscultation.*—"The information," we cannot forbear quoting the entire passage, "derivable from this mode of investigation is often nearly equally decisive, and is generally attainable at an earlier period than that dependent on percussion. But the sources of this information are also more variable; and require more delicacy, and a nicer discrimination, than that resulting from the comparative dulness of the two sides. In some cases, the only appreciable difference from healthy respiration is a decrease in the vesicular murmur. As this, however, sometimes naturally exists of different intensity on the two sides of the same individual, it is available, as a diagnostic sign, only when it is very decided, and when it co-exists with others which are of a less questionable character. In other instances, combined with the decreased vesicular murmur, there is heard, in one of either the infra-clavicular, acromial, or scapular regions, a marked increase of the sound of expiration; which in the natural state is nearly inaudible; and which, though it may not be particulary striking when attention is directed only to one part of the chest, becomes sufficiently obvious when compared with that observed in healthy portions of the lungs. In other, perhaps more advanced cases, in addition to the deficiency of the pure vesicular murmur, and to the increase of the expiratory sound, there exists a hoarseness of the respiration,—a diminutive, if I may be allowed the expression, of bronchial respiration. Andral states, that an increase of the natural respiratory murmur—and Dr. Stokes, that puerile respiration—is sometimes observable in the very portion of the lung which is affected with incipient phthisis. This statement may be correct; and it would be far from becoming in me to doubt the accuracy of such justly celebrated observers. I may still be allowed to say, that after considerable attention to the local signs of phthisis and other diseases of the chest, I have never yet had reason to think that pure puerile respiration results as an immediately local effect of either bronchial irritation preceding the deposition of tubercles, or of tubercular consolidation itself. It may indeed be, that the hoarseness of the respiration, of which I am now speaking, is that to which these authors refer. The respiratory sound is indeed louder than in the natural state, and no rattle is combined with it; but it is not, as it appears to me, simply an increase of the pure vesicular murmur, but resembles, as I have already said, a diminutive of bronchial respiration; and is produced, I doubt not, by thickening, or compression of the minute bronchial tubes. As milliary granulations are generally preceded by, and rarely exist in considerable numbers, without some bronchitis, there is not unfrequently added to the signs already mentioned, and sometimes obscuring the more delicate among them, either a small thin mucous rattle, resulting from the admixture of air with the glairy fluid existing in the minute tubes, or a single clicking sound, arising apparently from portions of thick viscid mucus, presenting an obstacle to the passage of air through those of larger calibre. These are not sometimes heard, on a superficial examination; and become audible only at the termination of a deep inspiration, and when the superficial parts of the chest are made tense by drawing the shoulders forcibly backwards, if the examination is made at the anterior, and by crossing the arms and inclining the body forwards when it is made at the posterior part. Two other signs afforded by auscultation may be mentioned; though I believe they are usually not so early in their appearance as those already referred to: they are, a trembling vibration of the voice, or a slight modified bronchophony, and an increased distinctness of the sounds of the heart observable over the affected portion of the lung; when the heart itself, and the great vessels connected with it, are ascertained, by previous or subsequent examination, to be in a healthy condition.

The whole of the preceding auscultatory signs—with the exception of the two last, which result from a partial consolidation of the lung—appear to depend upon thickening, or compression of the smaller bronchial tubes, or upon a secretion from their lining membrane; and may therefore all exist in simple bronchitis. From it, however, incipient phthisis may generally be distinguished, by the signs being confined to a small portion of the lung, which, in a vast majority of cases, and, in the simple forms of the disease, perhaps nearly universally, is the apex of the organ,—while bronchitis almost as commonly affects the entire surface of the bronchial membrane; by the continuance of the local signs of the former complaint, without important change; and by the general history and character of the disease. That the physical diagnosis cannot be always satisfactorily established, has been already acknowledged at the commencement of this paper."

It will be observed that the information given to us by Dr. Hughes coincides pretty nearly with what we have generally counted on—that the respiratory sound is often slightly increased and modified in the earlier stage of phthisis. It matters little what precise term is given to the alteration—*that* is too vague to allow any term to fit it without risk of misconception—and the result of our experience is, that the sign itself must be received with caution, and not depended on over much. Auscultators too frequently make sad blunders.

*Percussion.*—" The sign afforded by this mode of investigation, though late in its appearance, is perhaps more valuable than most of those that had been before mentioned as an indication of the *presence* of tubercles. Like them, however, dulness on percussion derives its importance, as a diagnostic, entirely from the part of the chest in which it is discovered. Thus, the same amount of dulness in the base and apex of the lung would be very differently regarded by the experienced auscultator. The one might be thought to result from pleuritis or pneumonia; the other, particularly if confined to one side, would be considered indicative of phthisis. I have almost always found that mediate is preferable to immediate percussion, and that the very best plessimeter is one or more fingers of the left hand. I have also found that minute differences of sound are best elicited by gentle percussion; and that they are more clearly distinguished immediately below the clavicles, than, as is recommended by many authors, by tapping the clavicles themselves. It is desirable, though I do not recollect to have ever met with or heard the observation, that the examination by percussion should be made during forced inspiration, and forced expiration, as well as during the ordinary degree of expansion of the chest; as, by these means, consolidation, even when separated from the surface by an intervening portion of healthy lung, may be sometimes discovered. When percussion has been performed on one side, the resulting sound should be immediately compared with that elicited by the same means from an exactly corresponding situation on the opposite side, and the strokes alternately repeated in quick succession. By adopting this procedure, slight differences of sound may be often made sufficiently obvious, which would otherwise have escaped detection. If both infra-clavicular regions sound well, and equally well, it is desirable to make a similar examination of the superior parts of the mammary regions; as it sometimes, though unfrequently, happens, that tubercular deposit first occurs in the latter. It is also desirable to examine by percussion the acromial and scapular regions; though I believe that the sounds afforded by them are not, in the healthy condition, sufficiently clear to indicate slight alterations of structure in the pulmonary tissue. If both infra-clavicular regions are equally though not sufficiently resonant, the sound should be immediately compared with that afforded by percussion of other regions, particularly the mammary, the axillary, and the lateral."

All this is judicious—*is* usually practised by careful and by well-informed men—*ought* to be practised by all.

Dr. Hughes adds :—

"If then, in a person labouring under bronchial irritation, with either a scrofulous constitution, or an hereditary predisposition to phthisis, there be observed a flattening or deficient expansion of one of the infra-clavicular regions,—if there exist permanent feebleness or hoarseness of the respiration, or an increase of the respiratory sound,—if a slight muco-crepitating, or small mucous rattle be discovered in the infra-clavicular, acromial, or scapular regions of one side (or indeed of both sides, when they do not exist in other parts of the lung), even though there should be no dulness on percussion,—the commencement of phthisis may, I feel assured, be predicted with considerable confidence. It is not probable, and indeed is scarcely possible, that all these signs should be present in the same individual at the same time ; though many may co-exist, and all may successively appear. The greater their number, and the more obviously marked their character, the less hesitation will there be in pronouncing an opinion upon their cause. Dulness or difference of sound, on percussion, if it should not be already distinguishable, will probably too soon make its appearance, unless prevented by appropriate remedial measures ; and high probability will then be converted to almost a certainty."

We have given these remarks, because they embody all that can be said upon a very interesting subject. We cannot flatter our author by professing that we discover any novelty in his mode of looking at it, or any suggestions which wander from the track of the experience of the best informed physicians. But, in our science, it is very useful to dish up a rechauffé of things which ought to be remembered, and are frequently forgot. And he who cooks the hash, or he who serves it may receive some thanks—as comfort for Dr. Hughes and for ourselves.

---

### VII. DR. GUY ON THE PULSE IN PHTHISIS PULMONALIS.

Dr. Guy, as our readers well know, has already given us some interesting observations on the pulse ; he has followed them up in the case of phthisis, and communicates them to the profession in the present number of the Reports. We extract his summary :—

"1. In cases of phthisis pulmonalis, the frequency of the pulse varies within wide limits ; the difference between the extremes amounting to 90 beats.

2. In the same individual, the frequency of the pulse undergoes remarkable fluctuations ; passing, in a few days, through a range of upwards of 60 beats.

3. In five out of six cases, the frequency of the pulse in phthisis exceeds the highest frequency observed in health.

4. The difference between standing and sitting in phthisis is nearly the same for all frequencies of the pulse.

5. The maximum difference between standing and sitting, in all cases of phthisis pulmonalis, falls short of the mean difference in health.

6. From the average results of a considerable number of cases, it appears that the mean difference in health is six times as great as the mean, and three times as great as the maximum, difference in phthisis.

7. On the supposition that the slight effect produced by change of posture is peculiar to phthisis pulmonalis, it forms one of the most constant and certain of its symptoms.

8. On the supposition that the slight effect produced by change of posture is common to more than one disease characterized by increased frequency of pulse, it will distinguish these diseases from others with which they may be confounded ; and these diseases themselves are easily distinguished from phthisis pulmonalis, either by the peculiar character of the pulse, or by other physical signs.

### VIII. Mr. Bransby Cooper on Amputation, &c.

Mr. Cooper has made some very interesting observations upon amputation. We extract the following:—

1. "Amputation may certainly be looked upon as one of the most formidable operations in surgery, not merely from its being attended with great suffering and danger to the patient, but also leaving him the subject of mutilation for the remainder of his life. I have been lately informed, that, in Paris, not more than three or four patients out of a hundred live after the operation of amputation: this, however, certainly, does not depend upon any want of skill in its performance by the French surgeons, but from their mode of after-treatment, and principally from their use of charpie, preventing the union by adhesion; leaving the healing of the stump to the granulating process, which is not only infinitely more tedious, but likewise calls much more upon the powers of the constitution.

The diet of the hospitals in Paris is not, as in our public institutions, under the regulation of the surgeons, but of the Government; and the use of stimulants and generous diet is pertinaciously forbidden, excepting in very urgent cases, when, perhaps, a little *vin ordinaire* may be allowed,—a beverage quite insufficient to maintain the powers of a patient under the protracted cure of a large wound by granulation.

To these causes, I believe, is to be attributed the want of success in the result of surgical operations in Paris; as, in dexterity of manipulation, and boldness in its application, the French surgeons are not to be surpassed."

2. "About six years ago, a patient was admitted, under my care, into Guy's Hospital, with a fracture of the lower third of the thigh-bone, from the kick of a horse. The fracture was oblique, and extended to within three inches of the knee-joint. The limb was placed upon a double-inclined plane, and a splint applied to either side of the thigh. On the fourth day after the accident, I discovered, for the first time, a diffused swelling in the ham, extending above the internal condyle, the swelling communicating a distinct pulsation. As the leg and foot retained their natural heat, and indistinct pulsation could be felt along the course of the posterior tibial artery, I considered there were yet hopes of saving the limb, by putting a ligature around the femoral artery; which I performed without removing the patient from his bed; thus saving him the pain and consequent ill effects of being carried to the operating theatre; and had the happiness, not only of saving his life and limb, but of restoring the extremity to its natural function."

3. "From the result of my experience, I consider it as a most unfavourable case where muscle or fascia prevents the adaptation of the fractured ends of a bone; and have generally found such cases terminate unsuccessfully. When want of union arises from constitutional causes, it is often very difficult to ascertain the nature of the defect: for I have, on several occasions, witnessed a great difficulty in producing the union of a fractured bone, in those who seem to be performing every function naturally, and in whom there was not a single symptom indicating the preference of one remedy beyond another. In such cases, I have, in three or four instances, found the administration of mercury, to an extent to produce ptyalism, of the greatest benefit: and have succeeded in producing consolidation, after seton, pressure, and other violent means, had failed. The efficacy of this practice is well worthy of further investigation; as a permanent disunion of the bones of a lower extremity must lead to the necessity of amputation; and if of an upper extremity, it would at any rate render the limb useless for the remainder of life."

4. "A young man, aged 18, four or five days prior to his admission, had, while bathing, trodden upon a nail, which had penetrated deeply into the sole of the foot. The puncture at the time produced but little pain; so little, indeed, that he continued in the water for some time after, and walked home, unmindful of the injury he had sustained: in a few days, however, the foot became painful and inflamed, and he thought he experienced some little difficulty in the movements of his jaw. Becoming alarmed at these symptoms, he applied at Guy's Hospital for relief; and on my attention being drawn to him, the symptoms of lock-jaw were evident. Purgative enemata were ordered; a grain of opium, and two grains of calomel, every three hours; and blisters to be applied, in the course of the spine. On the next morning, the report was, that all his symptoms were much relieved: the motion of his jaw was freer; he could swallow with less difficulty; and the injections had produced free evacuations from his bowels. Gratified with the evident mitigation of his sufferings, I too hastily formed a favourable prognosis; for, on desiring the patient to turn round in his bed, that I might examine the effect of the blister, he was seized with convulsion, and died instantly."

### IX. CASES OF HERNIA. By BRANSBY B. COOPER, F.R.S.

The following Cases possess some features of interest for every practical surgeon.

*Case* 1. On the 26th April, 1839, Mr. Frederick Toulmin was called to Mr. J. aged 68, who was suffering much pain in his bowels and at the pit of his stomach, attended with frequent vomiting; but represented, at the same time, that the bowels had been twice moved during the day. Mr. Toulmin, however, being aware that his patient was the subject of rupture, inquired whether it was up; and, upon being informed that "all was right," considered the symptoms arose from an attack of indigestion. Next morning Mr. J. passed a well-formed motion, but still had some vomiting and pain. In the evening the urgent symptoms had returned. Mr. Toulmin now made a close examination:—

The right inguinal region, the side on which Mr. J——— had been for many years the subject of hernia and had worn a truss, presented the appearance of an unnatural degree of flatness, particularly obvious when compared with the opposite side; but which seemed attributable to the absorption of fat, from the continued application of the truss. The fulness on the left side, however, was found not to depend wholly on the accumulation of fat, as a small hernia could be felt protruding through the external abdominal ring, but which immediately returned under the act of its examination: there was still, however, to be distinctly perceived a greater fulness on the left than on the right side. Under these circumstances, Mr. Toulmin ordered a pill, composed of calomel and rhubarb, to be taken immediately, and to be followed by a purgative draught in a state of effervescence; and a sinapism to be applied to the region of the stomach. On the 28th, the patient was much the same. On the 29th, the vomiting had become stercoraceous. On that afternoon, Mr. Cooper was called in.

"I first carefully examined the condition of both inguinal regions; having been informed that the patient had been the subject of double rupture; for many years on the right side, and for some months on the left. On taking a general view of the two sides, to ascertain the defect of symmetry, it was at once evident that the left was the larger of the two; not, however, presenting the appearance of a circumscribed tumor-like hernia, but more as if the prominence was the result of a natural deposition of fat, which had been absorbed on the right side from the pressure of the truss, and had thus led to the inequality of size. Upon manipulation, however, there was a somewhat unnatural sensation communicated, as well as a fulness in the left inguinal canal.

I then proceeded to examine the right side, and no protrusion whatever could be felt in the canal; but, upon desiring the patient to cough, the slightest exertion of the abdominal muscles immediately produced the descent of a hernia, but which was very easily returned into the cavity of the abdomen by the most gentle pressure. The fore-finger was then passed into the inguinal canal, by pushing the loose skin through the external ring; and then desiring the patient to cough, there was complete evidence of the absence of any descent through the internal ring; and therefore nothing here could be discovered to lead to suspicion.

Our consideration, therefore, was again directed to the left side, as being the probable seat of mischief; and its deviation from the natural appearance, slight as it was, could not but create suspicion, when taken in conjunction with the concomitant symptoms. We then agreed upon the propriety of exploring its condition. An operation was proposed to the patient, and acceded to: it consisted merely of laying open the left inguinal canal; in which was found a small empty sac, but not any thing which could in any way account for the symptoms; and we remained, therefore, in the same state of uncertainty as to the cause of the disease. The wound was dressed, and our patient was placed in bed. Some purgative medicine was considered advisable, and was ordered."

There was no material relief of the nausea, the constipation continued, and at 10 a.m. of the 30th, the patient died.

*Dissection.*—We first proceeded to explore the part subjected to operation; and found that the inguinal canal had been exposed; in which was seen the hernial sac, now containing a small portion of healthy omentum, which had descended probably since death. The sac was laid open, and its contents were most readily returned into the abdomen, not being subjected to the least constriction; —proving the correctness of the opinion formed during the exploration of the previous day, that on the left side there was no existing cause for the symptoms which had proved destructive to life. We then proceeded in our examination, by turning down the anterior parietes of the abdomen, for the purpose of carefully investigating the precise position of the intestines. Upon effecting this, there appeared, in the general view, nothing abnormal, either in their situation or degree of vascularity—not even at the left internal ring opposite to the seat of operation; although the portion of omentum which had descended into the hernial sac was lying close to the opening, but was distinguishable merely from the form which it had acquired from its frequent descent into the inguinal canal. There were, in this situation, however, no adhesions, no inflammation, nor any appearances to which could be attributed the functional disturbance. On examining the condition of the right inguinal region, the seat of the old reducible hernia, we found a portion of intestine in the sac, which proved as easily reducible as it had been during life; but on drawing the intestine out of the situation of the internal ring, it resisted displacement, as if some adhesions had retained it there. On examining the cause of this retention, it was found that a portion of intestine had become strangulated in a small hernial sac which was situated anteriorly to the larger one containing the reducible hernia. This portion of intestine, and its sac which strangulated it, were situated within the cavity of the abdomen; and could not have been relieved, even if the exploration had been performed on the right side; unless, indeed, the dissection had been performed on the hernial tumor while protruding into the scrotum; for which there could be no apparent reason, as the part was so easily reduced by the taxis. It is, however, to be admitted, that had the hernia been cut down upon, within the scrotum, without attempting its reduction, the anterior sac, with its strangulated intestine, would have been brought into view, and capable of being relieved, instead of being returned by the taxis into the abdomen in a state of constriction. But, as the fact of this second hernial protrusion could not be ascertained during life, no one, I consider, would have been warranted in such a proceeding: for if so, the taxis must at all times be considered as an improper practice, and every obstructed hernia must be subjected to operation.

It may however be learned, from this case, that when, after the reduction of a hernia by taxis, the symptoms of strangulation remain unabated, the tumor should, if possible, be made again to protrude, and then be submitted to operation; as it is evident its reduction has not relieved the constriction of the implicated viscus. This case is also highly important, as marking the necessity of a very close examination of the condition of a hernia, after the stricture has been divided external to the sac, before the contents of the sac be returned without the peritoneal covering being opened; for in such a case of complication as has been described, it is very possible that the contents of the larger hernial sac might be pushed into the abdomen, and the smaller sac with its strangulated intestine with it, if the neck of the sac were not opened for the division of the stricture.

*Case* 2.—I. A., aged 67, was admitted Nov. 18, 1834, with a strangulated oblique inguinal hernia on the right side. He said he had been the subject of hernia for thirty years; but never before experienced any difficulty in its reduction. On Monday morning, while at stool, his rupture descended, and he immediately became sick; when, upon being unable to push his rupture up, he became alarmed, and applied for admission into the hospital. He was then suffering from continual sickness; pulse soft and compressible; tongue dry and furred; skin cool, but clammy; countenance rather anxious. The hernia descended into the scrotum; but was not tender, even upon pressure. He walked to his bed with apparent ease: the reduction of the hernia being then attempted unsuccessfully, he was ordered into a warm-bath; and being subjected to its influence for half an hour, he became faint, and in so favourable a condition, that a further application of the taxis proved apparently effectual, for the hernia was returned into the cavity of the abdomen. On his return to his bed, he expressed himself relieved; and was anxious to have his truss applied, which was immediately done. A common enema was then administered; but it returned immediately, without producing any alvine evacuation. The urgent symptoms, however, soon returned; his pulse became quicker; he passed a most restless night, with occasional vomiting; and the constipation of the bowels remaind.

On the 19th, the obstruction was the same, and the matter vomited feculent. Mr. Cooper could discover no hernial protrusion. Mr. C. therefore ordered an injection to be given, by passing an elastic tube into the sigmoid flexion of the colon; hoping to be enabled to remove some hardened fæces, which might prove the cause of obstruction. Considerable difficulty, however, was experienced in the introduction of the instrument, and the injection was merely thrown into the rectum in the usual manner.

Next morning, an enema was again attempted to be thrown up; but he complained of so great a pain from the introduction of the instrument, that the operation was not satisfactorily completed; although two syringes' full of salts and senna were injected, but returned almost immediately. From this period he became much worse; his pulse and countenance sunk; and at half-past six he died.

*Dissection.*—Peritoneum generally inflamed. The two upper thirds of the small intestines were greatly distended, of a dull reddish hue, and covered with diffused arborescent capillaries: in the lower third, a knuckle of intestine, to the extent of about two inches, was found strangulated in a distinct sac, lying between the right linea ilio pectinea and the bladder, being placed in the natural pouch of peritoneum leading to the internal abdominal ring, and which usually forms the sac of an inguinal hernia. The bladder contained about five ounces of urine, and was peculiarly elongated upwards. The sac, as seen within the abdomen, seemed about as large in size as a large walnut: its aperture, which formed the stricture, was of considerable size, although the stricture around the intestine was pretty firm. The sac itself was not much discoloured, but the intestine contained within it was of a dark greenish hue. There was no-where any appearance of recent adhesions, or other solid effusions. The pelvis contained about two ounces of dull yellowish-red serum, of offensive odour. A firm and reddish margin of omentum adhered to the front of the sac very firmly, and some bridles of adhesive matter confined the cæcum in the iliac fossa. About three inches of the ileum, below the stricture, shewed evidence of former constriction; the middle part of this portion being slightly dilated, thickened, opaque, and injected; whilst the two extremities were marked by an irregular patch or flake, as if cicatrisation had been the result of some former physical compression. A preparation was made of the parts, and are preserved in the Museum of Guy's Hospital. The other abdominal viscera were sound; excepting the rectum, which, at the part corresponding to the promontory of the sacrum, presented a perforation surrounded by a dark ecchymosed spot, which had been evidently produced by the attempts to pass the elastic tube into the colon.

Mr. Cooper adds:—

"This case well exemplifies, that the return of a hernia into the cavity of the abdomen, by the application of the taxis, does not necessarily lead to the cessation of strangulation; and that all the symptoms of intestinal obstruction may remain, although there is no longer any abdominal protrusion. Such a condition offers great difficulty in the adoption of the treatment which is to be employed: the administration of purgatives cannot avail, as they tend only to increase the sickness, without offering any hope that they should overcome the mechanical obstruction. Enemata are perhaps indicated, as they may remove accumulated, hardened fæces, which might, indeed, be the sole cause of obstruction, and the hernia prove to be merely a coincidence: a suspicion of this fact possibly would arise, if the injections brought away a great quantity of scybalæ; after which purgatives might be safely used: but if these means prove ineffectual there is, I believe, nothing left to be done, but to have recourse to a surgical operation, which I should, under these circumstances, now never hesitate to recommend. The plan which I should adopt would be, first, to do all in my power to promote a return of the hernia; which, under the circumstances I have detailed, I think may very frequently be effected, as the effectual application of the taxis had already proved that the sac had formed no adhesions; and as soon as the swelling was reproduced, the sac should be cut down upon, and treated as in the operation for hernia generally: invariably however, in such cases, opening the hernial sac. But if, on the other hand, the hernia cannot be reproduced, then I consider the outlet of the abdomen, through which the protrusion had occurred, should be laid bare, and dilated, so as to facilitate the descent; or even, if necessary, so as to expose the hernial sac, and to enable you to divide the stricture. This operation, although dangerous and difficult, offers quite sufficient hope for success to render its adoption advisable; and is far better than to leave a patient to inevitable dissolution, if the obstruction be allowed to continue. Indeed, I will say, further, that the frequent attempts at reduction by the taxis, and the delay consequent upon these trials, are far more dangerous than the dexterous performance of the operation which I propose. It is, in fact, already granted, by all surgeons, that a patient with symptoms of strangulated hernia, and any concomitant abnormal enlargement about the abdomen, is a fit subject for operation. The exploration proposed, therefore, goes but one step further—to seek for the part, which, although returned within the abdomen, proves itself, in its present condition, incapable of performing its natural function."

